# Bi-Directional and Time-Lagged Associations between Engagement and Mental Health Symptoms in a Group Mindfulness-Based Mental Health Intervention

**DOI:** 10.3390/ijerph21081030

**Published:** 2024-08-05

**Authors:** Brock A. Rigsby, Reagan L. Miller, Megan J. Moran, Addie J. Rzonca, Jonathan I. Najman, Melanie S. Adams, Mark A. Prince, Rachel G. Lucas-Thompson

**Affiliations:** 1 Department of Human Development & Family Studies, Colorado State University, Fort Collins, CO 80523, USAlucas-thompson.rachel.graham@colostate.edu (R.G.L.-T.); 2 Department of Psychology, Colorado State University, Fort Collins, CO 80523, USA

**Keywords:** mindfulness, mindfulness-based intervention, mental health, psychological distress, daily diary, within-person variability, erraticism, location scale model, group intervention, university students

## Abstract

There is a high need for accessible avenues for improving mental health among emerging adults, particularly on college campuses. Mindfulness-based intervention (MBI) is a promising avenue for reducing mental health symptoms, but initial discomforts associated with MBI may cause symptoms to fluctuate before decreasing, which presents a barrier to engagement with mindfulness on a daily basis. Consistent mindfulness practice is key for forming habits related to MBI, and engagement with mindfulness at home, including between intervention sessions, is an important predictor of mental health outcomes. Research suggests that mental health symptoms may serve as barriers to their own treatment. Thus, it is important to understand how mental health symptom levels impact adherence to treatment protocols. To improve understanding of symptom-specific barriers to treatment and engagement with mindfulness, the present study collected daily diary surveys about engagement with mindfulness and mental health symptoms from a sample of 62 adults recruited to participate in a six-week mindfulness intervention. We explored mental health symptoms as a predictor of engagement with MBI at the mean level and whether within-person variability in symptoms predicted same-day or time-lagged changes in engagement via mixed-effects associations. Using heterogeneous location scale models, we further explored whether erraticism in either mental health symptoms or engagement with mindfulness predicted the other and if outcomes of the mindfulness intervention were homogeneous among subjects. Results showed that bi-directional and time-lagged associations exist between symptoms and engagement, indicating that there is a nuanced temporal and reciprocal relationship between engagement with mindfulness and mental health symptoms. Daily within-person elevations in engagement with mindfulness were associated with concurrent improvements in mental health but prospective increases in mental health symptoms. We also found that higher engagement (over personal averages) was not consistently associated with improvements in mental health across the sample but was instead associated with greater heterogeneity in outcomes. We also found that increases in mental health symptoms (over personal averages), as well as higher average levels of mental health symptoms, were both associated with lower levels of engagement in the mindfulness treatment protocol.

## 1. Introduction

Anxiety and depression are highly prevalent in adolescence and early adulthood. Studies prior to the COVID-19 pandemic estimated that more than half of American college students felt hopeless, overwhelmed, exhausted, lonely, sad, or anxious [1], and post-pandemic studies indicate that undergraduate students perceived significant increases in their own symptoms as a result of the pandemic [2]. These estimates indicate a high need for accessible mental health services at colleges and universities in the United States. Yet, high demand for clinical mental health services has outpaced the ability of colleges and universities to provide them, as university counseling center directors report being understaffed [3]. This pattern highlights the need for targeted and accessible mental health interventions, such as mindfulness-based interventions offered via telehealth.

Mindfulness-based intervention (MBI)—including a variety of approaches and formats—has been shown to significantly reduce symptoms of anxiety and depression through pre-post observational [4], quasi-experimental [5], and randomized control trial designs [6]. MBI is feasible, acceptable, and efficacious for this age group [7,8], and meta-analytic evidence suggests that MBI with university students, compared to other populations, may yield larger effect sizes [6]. Thus, MBI is one possible avenue for delivering the necessary accessible mental health intervention. However, effect sizes associated with MBI are often small to moderate [4]. Initial discomfort associated with mindfulness practice—which may increase mental health symptoms [9] contrary to the goals of MBI—may contribute to these effect sizes by reducing engagement and likelihood of daily practice, which are key contributors to positive outcomes of MBI [10,11,12]. Further, some mental health symptoms have been shown to reduce engagement with intervention [13,14]. Thus, it is important to understand how engagement and symptoms interact at a daily level.

### 1.1. Treating Anxiety and Depressive Symptoms with MBI

Mindfulness—which can be trained in individual or group settings, by clinicians or non-clinicians, and virtually or in-person—is positively associated with psychological health, such that mindfulness training (e.g., through MBI) leads to lower levels of mental health symptoms (e.g., depressive symptoms, anxiety) [6,15,16,17,18]. The mindfulness stress-buffering hypothesis suggests that more mindful people are able to reduce their physiological response to stress by forming less negative appraisals of stressors [11]. Thus, it is important to note that MBIs are unlikely to focus on reducing or suppressing levels of experienced stress [19]; rather, they train mechanisms through which the effects of stressful experiences can be mitigated [9,11]. Training mindfulness skills, such as present-moment awareness and non-judgmental appraisals of experiences, takes time and practice [20]. In some situations, MBI is uncomfortable at first [9,21], which may increase symptom levels prior to improving them [9,12,22]. However, this discomfort wanes over time as familiarity with mindfulness increases [9,21].

Consistent and long-term engagement with mindfulness practice has received significant support as an efficacious treatment option for a variety of mental health issues [10,11,12,23]. Even smaller doses of mindfulness are effective in symptom reduction [24], but effect sizes are smaller compared to longer-term mindfulness practice. Thus, a lack of adherence to MBI recommendations for practice between sessions, likely to be characterized by bursts of smaller doses and high levels of variability in the amount of daily practice—with less or slower waning of initial discomfort—seems unlikely to afford the benefits of consistent mindfulness practice. Engagement with treatment protocols both during and outside of treatment sessions is an important marker of adherence to recommendations. Completion of “therapy homework”, for example—an indicator of engagement in treatment recommendations outside of psychotherapy sessions—may be inconsistent across weeks during treatment for anxiety [25]. Across disorders, though, including therapy homework in treatment significantly improves outcomes [26], and compliance with homework has small to moderate effects on treatment outcomes [27].

During the course of an MBI, between-session mindfulness practice is critical for the development of mindfulness skills. Gains in mindfulness over the course of an intervention are significantly and positively associated with between-session mindfulness [28]. In fact, time spent engaging in mindfulness during the course of an MBI is perhaps the most robust predictor of increases in well-being [29,30]. Further, the amount of formal mindfulness practice at home is negatively associated with relapse of major depression symptoms following intervention [31] and intended effects of treatment on mental health [29,32]. Although previous work suggests it is important to normalize inconsistent practice [33,34], consistent practice is necessary for forming habits related to mindfulness. Understanding the patterns and predictors of engagement at the daily level is, then, important in understanding how to optimize MBI to have the greatest impact on mental health symptoms. Yet, existing work has focused exclusively on mean-level assessments of engagement or total engagement across an intervention without focusing on daily patterns of engagement, which may have differential effects on outcomes. These findings highlight the necessity of at-home practice and the importance of researchers building an understanding of barriers to adherence to treatment protocols and recommendations between sessions, including, in MBI, consistent engagement with mindfulness between intervention sessions. Key barriers such as lack of goal-setting, limiting beliefs, and low self-monitoring, which inhibit mindfulness practice [13], are similar in nature to the symptoms of depression and anxiety. Because mental health and engagement with treatment protocols are likely to change day-to-day, it is important to evaluate engagement at the daily level.

### 1.2. Symptoms as Barriers to Treatment

Daily, stressful life events may occur. These often lead to emotional reactivity and increases in mental health symptoms, especially among individuals with a history of mental health disorders [35]. Further, a manifestation of symptoms may negatively impact adherence and engagement with treatment protocols [13,14,36]. To effectively adapt accessible interventions for college students, specifically, it is important to understand nuances of their stressors, mental health symptoms, and patterns of symptom fluctuation. These fluctuations in symptoms may impact engagement with treatment protocols, especially between treatment sessions. Given that between-session engagement in mindfulness practice is key in supporting increases in mindfulness, formation of habits related to mindfulness practice, and improving mental health [13,28], understanding the interactions between symptoms—especially those that are the target of treatment—and participants’ ability to engage with intervention protocols is one under-studied facet of MBI capable of boosting treatment effects.

There is precedent for the idea that mental health symptoms may interfere with or otherwise impact participants’ own treatment. Anxiety and depressive disorders are often comorbid [37,38,39] and share features like fatigue, restlessness, difficulty concentrating, and sleep disturbances [40,41]. These shared symptoms, in addition to disorder-specific symptoms such as loss of interest/pleasure, irritability, temper, feelings of hopelessness, hesitation or refusal to leave home, and fear/anxiety about social situations [40] may serve as barriers to treatments designed to target similar symptomatology. For example, social anxiety may prevent attendance in community mental health interventions [42] and fear that new time commitments will exacerbate existing levels of stress may lower the desire to begin or fully participate in intervention [21]. However, the body of empirical literature on symptoms and symptom fluctuation as treatment barriers is relatively underdeveloped.

Anhedonia—a common symptom of depression, characterized by loss of pleasure or interest in typical activities—has received recent attention as one specific barrier to motivation and engagement in depression treatment [14]. Khazanov et al. (2022) highlighted the need for anhedonia symptom-specific treatment in order to improve engagement with and outcomes for depression treatment [14]. Illustrating a significant need for development in this literature, Khazanov and colleagues (2020, 2022) hypothesize that anhedonia’s effects on motivation and engagement in treatment may have causal effects in the established negative association between anhedonia symptoms and desired treatment outcomes [14,43]. Further understanding of symptom-specific treatment barriers will better inform mental health providers in clinical and community settings as they design mental health treatments that are effective despite the targeted mental health challenges of participants.

### 1.3. The Present Study

In the present study, we explore the effects of mental health symptoms on engagement with between-session treatment protocols—specifically, daily practice of trained skills—in an MBI. Patient adherence to treatment protocols is a critical factor in achieving desired treatment outcomes. Factors contributing to indices of treatment adherence, such as dropout, have been explored, and several therapist-, client-, and treatment-related variables have been ruled out as significant predictors [44,45]. However, a significant gap in the literature related to symptom-specific barriers to treatment and engagement exists. Theoretical literature related to this topic has called for an examination of the effects of symptom-specific barriers on treatment initiation and continuation, as well as levels of engagement [14]. The present study seeks to address this gap by exploring the effects of symptoms of anxiety and depression on adherence to treatment protocols, operationalized in this study as engagement in mindfulness practice at the mean level, and whether daily within-person changes in symptoms have immediate or time-lagged effects on engagement with treatment. We expected to find that symptoms serve as a barrier to their own treatment such that higher mean levels of symptoms were associated with lower engagement with treatment and that within-person fluctuation in symptoms impacts concurrent (same day) and prospective (next day) engagement with treatment.

## 2. Materials and Methods

### 2.1. Participants

Data for the present study were derived from a parent study (total *n* = 62, pre-/post- analytical sample *n* = 50) [46]. Students at a large public university in the northern part of the U.S. state of Colorado (*n* = 62) were recruited from university class lists, psychological services waitlists, and participant pools to participate in a six-week mindfulness group program called Learning to BREATHE [47], and a study to test the effects of that program.

### 2.2. Procedure

All procedures were approved by the Colorado State University Institutional Review Board. Interested participants, recruited through undergraduate class lists and the waitlist of an on-campus mental health center, completed an online screener to determine eligibility. To be eligible, participants were required to own a smartphone and report “occasional”, “frequent”, or “very frequent” symptoms of anxiety or stress in their daily life. Eligible participants provided verbal and electronic informed consent to participate in the group intervention program and study. Participants in the parent study were randomized into one of five groups, each with differing technological supports (e.g., encouraging text messages, activity libraries) between intervention sessions [46]. All condition groups have been combined for this study, which evaluates patterns in daily diary reports rather than condition differences or overall program effectiveness to increase statistical power and in the absence of hypotheses about condition differences. Only procedures relevant to the present study are presented here.

Study data, including demographic variables, were collected and managed using Research Electronic Data Capture (REDCap) hosted at the University of Colorado at Denver [48]. Subjects participated in a mindfulness program that included six units (one per week) focused on the following themes: the body, reflections (thoughts), emotions, attention, tenderness, and healthy habits of mind. Throughout the program, participants completed daily diary surveys and ecological momentary assessments (EMAs) delivered to their smartphones via the mobile application TigerAware [49]. At the end of the program and study, participants were compensated for their engagement with daily diaries and EMAs. Participants were not compensated for attending weekly intervention sessions.

### 2.3. Measures

#### 2.3.1. Daily Mental Health Symptoms

Daily Psychological Distress. As part of their daily diaries, participants completed the 4-item version of the Perceived Stress Scale (PSS) [50] to provide self-reports of psychological distress. Items were modified to gather daily perceived stress and were rated from 0 = “Never” to 4 = “Very often”. Example items include, “Today I felt unable to control the important things in my life” and “Today I felt things were going my way”. Positively worded questions were reverse-scored so that higher scores reflect more stress. Then, scores were averaged across items to create a total daily score of perceived stress (*M* = 2.55, *SD* = 0.35).

Daily Depressive Symptoms. Participants reported their daily depressive symptoms by responding to five items such as “Did you feel sad, hopeless, discouraged?” and “Did you feel exhausted, worn out?” Responses were on a scale from 1 = “Not at All” to 10 = “Extremely”. Scores were averaged to create a total daily score of depressive symptoms (*M* = 4.97, *SD* = 1.67).

Composite. To create a composite “daily mental health symptoms” variable, we standardized psychological distress and depression scores provided in daily diaries. Then, we averaged these standard scores to represent overall daily mental health (*M* = 0.00, *SD* = 0.91). The creation of this composite variable was necessary due to high levels of comorbidity and, thus, collinearity between psychological distress (anxiety) and depression [51,52,53].

#### 2.3.2. Daily Engagement with Mindfulness

To assess participants’ level of engagement with mindfulness practice outside of the group program, each day, participants were asked, “Today, how many mindfulness practices have you completed?” (*M* = 2.02, *SD* = 1.98). “Practices” in the Learning to BREATHE program include smaller, informal practices. Answers were provided by manually typing a number into the response field. Responses ranged from 0 to 33 (*M* = 1.97, *SD* = 1.90).

#### 2.3.3. Control Variables

As part of the baseline survey, participants provided their numerical age. They also completed the Mindful Attention Awareness Scale-Adolescent Version (MAAS-A) to indicate their baseline level of mindfulness [54]. The MAAS-A includes 14 items like “I do jobs or tasks automatically, without being aware of what I’m doing”, and participants indicate their level of endorsement of each item using a scale from 1 = “Almost Always” to 6 = “Almost Never” (α = 0.83).

### 2.4. Data Analysis Plan

Analyses were conducted in Stata version 15.1 [55]. Linear mixed-effects models were employed to explore concurrent (same-day) relations between mental health and engagement with mindfulness. Using daily composite scores, we calculated for each participant a decomposed stable average of mental health symptoms and engagement across the study (i.e., a person’s average across all repeated measurements), which represent between-person differences. We computed daily variability (within-person) in mental health symptoms (*SD* = 0.24) and engagement (*SD* = 5.05) by calculating the difference between the individual’s stable average and the daily diary score for each day of the intervention. Then, time-lagged variables were created for daily engagement with mindfulness and daily mental health symptoms.

Using multivariate mixed modeling (of random intercepts and slopes), we evaluated whether daily within-person variability in engagement predicted prospective (next-day) mental health symptoms, controlling for concurrent mental health and prospective engagement. Then, using a second multivariate mixed model, we evaluated whether daily within-person variability in mental health symptoms predicted prospective engagement, controlling for concurrent engagement and prospective mental health symptoms. To evaluate whether erraticism (inconsistency) in engagement with mindfulness impacted daily mental health and whether erraticism in mental health symptoms impacted daily levels of engagement, we employed mixed-effects location scale models [56]. These models were fitted using the *runmixregls* program within Stata [57]. Due to this study’s focus on intraindividual variability in a relatively small sample, demographic characteristics were not included as potential confounds. All models did, however, control for variables found to predict the missingness of daily diary data (age and baseline mindfulness).

## 3. Results

### 3.1. Preliminary Analyses

Participants were between 18 and 43 years old (*M*_age_ = 21.85, *SD* = 4.69). Table 1 presents other demographic characteristics of the sample. Participants in the program attended an average of 2.78 sessions (*SD* = 2.38). In total, 59 participants provided 1184 daily diary reports (*M* reports = 20.10, Range = 1–36) of psychological distress, depressive symptoms, and engagement with mindfulness. Of those, 58 participants provided 877 reports on consecutive days without “breaks” (*M* consecutive reports = 15.1, Range = 1–34), allowing for the creation of prospective variables and time-lagged analysis. Based on Monte Carlo simulations for statistical power in two-level models presented by Arend and Schafer [58], power in the present study was adequate to detect small-to-moderate level one direct effects in our daily and next-day mixed models.

To examine missingness in daily diary data, we tested the extent to which demographic and baseline mental health variables predicted the number of daily diaries participants completed. Age (−) and baseline mindfulness (+) were significant predictors of the number of daily diaries completed, such that younger and more mindful participants were more likely to submit daily diary surveys. So, these variables were included in all primary analyses as controls. No other variables were significantly associated with the number of daily diaries completed.

In addition to controlling for age and baseline mindfulness, we completed sensitivity analyses related to condition group assignment. The direction and significance of all findings replicated in these analyses controlling for group assignment. Therefore, to maximize statistical power, and given the lack of evidence to suggest that group assignment affected patterns of intraindividual variability—which were the key focus of this study—we present only the most parsimonious models in the tables.

Table 2 provides descriptive information about mean-level associations between average daily mental health symptoms and average daily engagement with mindfulness across all participants and all days for which scores were available. On average, across the intervention, mental health symptoms were negatively associated with engagement, such that greater levels of mental health symptoms were related to lower engagement with mindfulness practice.

### 3.2. Primary Analyses

#### 3.2.1. Time-Lagged Effects of Mental Health on Engagement with Mindfulness

Using a multilevel mixed effects model, we tested the prospective effects of daily mental health symptoms on engagement with mindfulness (controlling for concurrent engagement and prospective mental health). Average mental health symptom level was not a significant predictor of prospective engagement with mindfulness (Table 3). However, within-person deviation in mental health symptoms on the first day was significantly and positively associated with prospective engagement (Table 3), such that on days when individuals reported higher levels of mental health symptoms (relative to their own stable average of mental health symptoms) they also reported engaging in more mindfulness practice on the next day. In addition, we found a significant prospective association such that engagement on one day was a positive predictor of engagement on the next day. Finally, there was a negative concurrent (next-day to next-day) association between mental health and engagement, such that greater symptom levels predicted lower levels of engagement with mindfulness, which is consistent with preliminary between-persons findings presented in Table 2.

#### 3.2.2. Time-Lagged Effects of Engagement with Mindfulness on Mental Health

Using a multilevel mixed effects model, we tested the prospective effects of daily engagement with mindfulness on mental health symptoms (controlling for concurrent mental health and prospective engagement with mindfulness). The average level of engagement with mindfulness was not a significant predictor of prospective mental health (Table 4). However, within-person deviation in daily engagement with mindfulness was a significant and positive predictor of prospective mental health symptoms (Table 4), such that on days when individuals reported greater engagement with mindfulness (relative to their own stable average of engagement), they also reported higher levels of mental health symptoms on the next day. In addition, we found a significant prospective association such that mental health on one day was a positive predictor of mental health symptom levels on the next day. Finally, there was a negative concurrent (next-day to next-day) association between engagement and mental health, such that greater engagement with mindfulness predicted lower mental health symptom levels, again consistent with preliminary between-persons findings (Table 2).

#### 3.2.3. Erraticism in Relation to Concurrent Symptoms and Engagement

Next, we fit a mixed-effects location scale model to determine the extent to which mental health symptoms predicted between-person (homogeneity vs. heterogeneity) and within-person (consistency vs. erraticism) variability in engagement in mindfulness practices. There were two significant negative associations—between daily mental health symptoms and heterogeneity in engagement, and between the average level of mental health symptoms and heterogeneity in engagement (Table 5, “between-subjects variance”)—such that participants were more similar to each other in having low levels of engagement with mindfulness (see Table 2) when they are experiencing elevations in mental health symptoms (relative to their own average), or when they have poorer mental health on average. Although erraticism in mental health symptoms was not significantly associated with variability in the engagement at the within-person level (Table 5, “within-subjects variance”), we did find a negative within-person (consistency vs. erraticism) trend between the average level of mental health symptoms and engagement with mindfulness such that people with poorer mental health may be more erratic in their engagement with mindfulness. This model also revealed that there was significant variability in the consistency of engagement in mindfulness practices from day to day (Table 5, “scale”) and that participants with higher mean levels of engagement with mindfulness were significantly more erratic, or less consistent, in their levels of daily engagement across time (Table 5, “association”).

Then, we fit a second location scale model to evaluate the extent to which engagement in mindfulness practice predicted between-person (homogeneity vs. heterogeneity) and within-person (consistency vs. erraticism) variability in mental health symptoms. Engagement significantly and positively predicted between-person heterogeneity in symptoms (Table 6, “between-subjects variance”), such that on days when participants engaged in more mindfulness practices (relative to their own average), participants were less similar to each other in terms of having fewer mental health symptoms (see Table 2). Again, there was no significant association between daily engagement and erraticism of symptoms (Table 6, “within-subjects variance”). In addition, although there was significant variability in the consistency of mental health symptoms from day to day (Table 6, “scale”), there was no significant association between mean levels of mental health symptoms and consistency of symptoms across time (Table 6, “association”).

#### 3.2.4. Erraticism in Relation to Prospective Symptoms and Engagement

Finally, we fit two location scale models mirroring those presented in Table 5 and Table 6 but testing prospective outcomes. The first, which tested the effects of daily mental health on prospective engagement with mindfulness, revealed a significant and positive association between intraindividual deviations in mental health and heterogeneity in prospective engagement with mindfulness, such that participants were more similar to each other (in terms of having low levels of engagement with mindfulness) on the day after their mental health was poorer than typical (α = 0.47, SE = 0.17, *p* = 0.006, 95% CI [0.13, 0.80]). However, we did not find any significant within-person (consistency vs. erraticism) associations. The second, which tested the effects of daily engagement with mindfulness on prospective mental health, revealed only a significant within-subjects association between mean levels of engagement and erraticism in next-day mental health, such that individuals with higher mean levels of engagement with mindfulness had more erratic (less consistent) prospective mental health symptoms (τ = 0.20, SE = 0.09, *p* = 0.017, 95% CI [0.04, 0.37]).

## 4. Discussion

The goal of this study was to explore associations between within-person variability in mental health symptoms and engagement in mindfulness practice (a part of the mental health treatment protocol in the context of a mindfulness-based intervention). We found that individuals experiencing higher levels of mental health symptoms (i.e., at greater need for treatment) showed lower engagement with the treatment protocol (i.e., daily mindfulness practice), on average. Individuals experiencing within-person elevations in mental health symptoms were less likely to engage with mindfulness during their time of greatest need for intervention. Higher engagement with mindfulness, compared to personal average, predicted increased mental health symptoms on the next day. Together, these findings suggest a bidirectional feedback loop between mental health symptoms and engagement with mindfulness practices. The study contributes crucial information regarding the time-lagged effects of engagement with mindfulness practice to the existing literature. Further, it expands the understanding of reciprocity between mental health symptoms and engagement with mindfulness as assigned during a treatment protocol.

Perhaps the most significant contribution of this study is that concurrent (same-day) and prospective (next-day) effects were distinct from each other. Increases in daily engagement with mindfulness were related to fewer concurrent mental health symptoms but consistently higher levels of prospective mental health symptoms. Subjects were similar (homogeneous) in responding this way. In turn, higher levels of mental health symptoms predicted less concurrent engagement with mindfulness but more prospective engagement. Again, subjects were similar in this pattern of response. These findings suggest that there is a complex pattern of day-to-day interaction between symptoms and engagement, which should be explored by future empirical research. Positive effects of mindfulness on concurrent mental health, which are well-established [11,12,24,59], were further supported by this study’s same-day findings. However, past work in this area has largely failed to consider within-subject time-lagged associations, which are important in understanding the non-immediate effects of mindfulness practice [60,61]. In addition, this study’s findings suggest that participants experiencing fewer mental health symptoms may be more likely to practice mindfulness concurrently than their more psychologically distressed peers. Those distressed peers may be less motivated, resourced, or inclined to practice new healthy habits of mind during their most distressed moments. Conversely, increased engagement with mindfulness was associated with better concurrent mental health. Taken together, these findings provide support for the hypothesis that participants’ psychological symptoms may become a barrier to their own treatment and that concurrent mental health intervention may be least accessible to people with the highest need. Past work related to symptom-related barriers to treatment has been extremely limited and specific to certain clinical symptom presentations. For example, past work has indicated that social anxiety has a similar effect on its own treatment [42]. Other, more recent work has focused on anhedonia (inability to feel pleasure), which often decreases motivation due to lack of reward, as a barrier to the treatment of depression, finding that this symptom of depression can hinder the treatment of depression [14]. In this study, we have shown that psychological symptoms need not follow such a specific presentation to impact their own treatment. This finding suggests that clinicians and researchers alike should address a major gap in knowledge about symptom-related barriers to treatment, which may impact a wide variety of community and clinical presentations.

Past findings in support of the efficacy of MBI suggest the frequency of practice is the most important predictor of outcomes [29,30], which seems to indicate that simply increasing the frequency of practice on days with poorer mental health might lead to improvements in mental health, sit in contrast with this study’s results. Our findings that sudden increases in mindfulness practice (over and above one’s own average engagement) may lead to poorer prospective mental health would caution against such an approach. Initial discomfort with mindfulness-based intervention is expected and well-documented [9,12]. This discomfort is expected to wane over time as tolerance and understanding build for unpleasant emotions and experiences [12]. However, participants in this study showed inconsistency in symptoms and engagement across the study, which might decrease the likelihood that discomfort wanes over time to yield the expected benefits. The possibility of discomfort that leads to “backlash” in symptoms runs contrary to the goal of any mental health intervention. Thus, future research should explore the extent to which discomfort persists over time and to what extent this discomfort persists as a result of inconsistent engagement. This could be accomplished through in-depth longitudinal analysis of these variables at the daily or momentary level and could lead to the development of strategies to both improve consistent engagement with mindfulness and decrease the likelihood of delayed backlash in symptoms.

Individuals who experienced increased mental health symptoms on one day were more likely to practice mindfulness on the next day, even though they were unlikely to engage with mindfulness while experiencing that increase in symptoms. This pattern in temporal predictive ability suggests that practice may be “reactionary”; in other words, participants in our intervention tended to increase engagement with mindfulness in an attempt to buffer the distress they had experienced on the day before [11]. Although this increase in engagement likely represents a small dose of mindfulness among those with low mean levels of practice, even small doses of mindfulness may be an effective self-regulation strategy [24]. However, such reactionary practice may indicate that participants viewed mindfulness as an option for responding to symptoms rather than a habit to form [13], limiting true familiarity with and consistency in mindfulness practice at the daily level. This decrease in engagement following positive outcomes could delay or prevent the waning of initial discomfort with mindfulness [9,21], and could increase levels of some symptoms [12]. Taken together, this pattern of results suggests that increasing mindfulness in response to symptoms is not likely to have effects lasting to the next day, and individual mental health is likely to return to normal, or even decline, after exerting effort to increase practice. Thus, in order to have lasting effects, MBIs for internalizing symptoms may require tailoring to specific symptom manifestations [14], which is less feasible in group interventions than in traditional clinical approaches.

In addition, these findings provide insight into participants’ low motivation to continue to practice in order to build habits. As symptoms increase or return to normal, which may be due to an increased awareness of symptoms rather than a true increase in symptom level [9,12,62], participants were less likely to continue to engage with mindfulness. Regardless of the source of perceived increases in symptoms, people make decisions based on costs and benefits [63]. If the cost of mindfulness practice is cognitive and emotional fatigue, and the perceived benefit or outcome is either a return to baseline or a next-day increase in symptoms, participants might lose motivation to complete the task of practicing mindfulness [63]. This pattern might be especially prevalent in individuals with clinical levels of anxiety or depression, where low motivation is a likely outcome of symptoms. If a pattern of reactionary practice and poor perceptions of next-day benefits to mental health continues to emerge in future studies, it is critical to build capacity within MBIs to target the fatigue associated with mindfulness practice [62] and promote motivation to form habits. Motivation is a key factor in behavior change and is understood to be highly modifiable [64]. Such an approach would likely expand the population of people who could benefit from mindfulness practice and improve the long-term efficacy of practice for MBI participants. Empirical research evaluating the role of motivation in mindfulness practice revealed that dimensions of motivation (e.g., perceived competence, interest, value/usefulness) were predictive of attendance at MBI sessions and pre- to post-intervention changes in stress [65]. Development of strategies to supplement MBI with targeted motivational or self-efficacy messaging delivered either during MBI or via technological supplements (e.g., apps, text messaging) has been shown to improve outcomes of MBI in college-aged participants [46]. The present study highlights the necessity of extending this work to target motivation and variability in symptoms.

Similarly, though there is an existing, yet limited, literature documenting contraindications for mindfulness practice [12,66], literature identifying populations wherein mindfulness may be less effective or lead to higher levels of initial fatigue—even if not explicitly contraindicated—is scarce and should become a focus of future research. We found, for example, that intraindividual increases in engagement with mindfulness had significantly inconsistent (heterogeneous) effects on mental health across participants. The literature documents numerous potential moderators of the beneficial effects of mindfulness, including motivation [65], personality [67,68], gender [69], and age [70,71]. Although the present study only accounted for age in its analyses, this line of inquiry, centered on understanding for whom mindfulness consistently leads to improved mental health, could also apply to other intervention strategies and contribute to a refined understanding of which treatments are most effective for certain symptom presentations or populations. In addition to mean-level moderating effects, it will be important for future research to examine the role of moderation at the daily level—an evaluation for which this study provided a foundation—within diverse samples. It is important to note that this study was conducted with a primarily white female sample, which limits the generalizability of our findings significantly. Because this study focused on intraindividual changes in a relatively small sample, we did not control extensively for personal and demographic characteristics unless these characteristics were found to affect missingness. Future research with larger samples, however, should evaluate these characteristics (e.g., race, gender, baseline mental health symptoms) as potential confounds.

Finally, erraticism of engagement and/or mental health symptoms is one possible contributor to patterns of engagement, and it was explored in the present study. Interestingly, the most engaged participants also showed the most erraticism in their engagement. This pattern of engagement seems reasonable when interpreted alongside past findings that mindfulness presents new practitioners with high cognitive and self-regulatory costs [12,62]. Consistency, leading to habit formation, is clearly an important factor in generating long-term benefits from mindfulness practice [10,11,12], and initial discomforts from mindfulness practice—likely similar to effects of inconsistent practice and lack of habit formation—contribute to increases in mental health symptoms [9]. Research has found, for example, that in the absence of adequate opportunity for practicing mindfulness skills, self-regulatory resources were fatigued or depleted during a mindfulness task in the face of a stressor [62]. Reactionary practice patterns, as seen in our sample, are similarly ineffective as a response to mental health symptoms, as the skills were unlikely to be practiced during appropriate opportunities.

Although this study makes important contributions to our knowledge about mental health symptoms and engagement with a mindfulness intervention, there are important limitations to note. Future research should employ methods that expose time-lagged patterns over longer periods of time. This study was limited in its exploration of only next-day lags, whereas different patterns may emerge on days more distal to an initial change in mental health symptoms or level of engagement. Our analyses were further limited by a lack of daily-level understanding of individual experiences and emotions that arose during engagement with mindfulness and the perceived quality of that engagement. Items included in future work should directly measure experiences with engagement at the daily level (e.g., “How did you feel while you practiced mindfulness at home today?”) and perceived quality of engagement [72,73] to provide important context for the interpretation of complex time-lagged findings. On a broader scale, mindfulness research has lacked a consistent manner of evaluating the quantity of mindfulness practice, quality of mindfulness practice, and adherence to mindfulness-based protocols. These inconsistencies in measurement limit our ability to interpret means in this sample compared to other studies [72,73]. This sample could, for example, have had a low-quality engagement or low levels of engagement compared to other samples such that participants never reached a helpful level.

## 5. Conclusions

Exploration of mental health symptoms as a barrier to their own treatment is a crucial area for mental health research. This study indicated that treatment and intervention may be least accessible to the individuals who need it most, especially at the times when they need it. Although this study focuses on a community mindfulness-based group intervention, the findings provide directions for future research with clinical samples and other treatment approaches. Further, this study illustrated the need for mindfulness researchers to consider the time-lagged effects of mindfulness practice, especially among new practitioners. Time-lagged effects were distinct from same-moment effects and were in the opposite direction of mental health treatment protocol goals. Thus, existing work demonstrating the proximal benefits of mindfulness practice lacks a critical perspective related to more distal effects. Consideration of these distal effects can improve the development of mindfulness-based interventions. Finally, this study showed that within-person variability in both mental health symptoms and intervention engagement are key indicators of both mindfulness and mental health outcomes. Because symptoms and engagement are not consistent day-to-day, understanding the nuances within deviations and patterns of change may lead to improved efficacy of interventions.

## Figures and Tables

**Table 1:** Participant Characteristics.

Demographic Characteristics	%
**Gender**	
Female	64.4
Male	17.8
Non-binary, gender fluid, gender queer	7.7
Did not report	10.0
**Race**	
Caucasian	81.1
Asian/Pacific Islander	8.9
African American	4.4
American Indian	2.2
Other	2.2
Did not report	1.1
**Ethnicity**	
Hispanic or Latino	15.6
Not Hispanic or Latino	74.4
Did not report	10.0

**Table 2:** Mixed-effects associations: mental health symptoms and engagement with mindfulness.

Effect	*b* (SE)	*p*	95% CI
Daily Engagement			
(Intercept)	2.02 (0.17) ***	<0.001	[1.68, 2.35]
Daily Mental Health	−0.20 (0.06) **	0.001	[−0.31, −0.08]
Daily Mental Health			
(Intercept)	0.09 (0.08)	0.275	[−0.07, 0.25]
Daily Engagement	−0.04 (0.01) ***	0.001	[−0.07, −0.02]

*** *p* < 0.001, ** *p* < 0.01.

**Table 3:** Mental health predicting prospective engagement with mindfulness.

Next-Day Engagement	*b* (SE)	*p*	95% CI
(Intercept)	2.19 (0.97) *	0.023	[0.30, 4.08]
M_Mental Health	0.13 (0.06)	0.571	[−0.32, 0.58]
DEV_Mental Health	0.20 (0.06) **	0.001	[0.08, 0.32]
Same-Day Engagement	0.25 (0.03) ***	<0.001	[0.19, 0.32]
Next-Day Mental Health	−0.24 (0.06) ***	<0.001	[−0.36, −0.12]
Age	−0.02 (0.03)	0.413	[−0.08, 0.03]
Baseline Mindfulness	−0.50 (1.24)	0.689	[−2.93, 1.94]

M_Mental Health represents the decomposed stable average of daily mental health symptoms, and DEV_Mental Health represents intra-individual variability in daily mental health symptoms. *** *p* < 0.001, ** *p* < 0.01, * *p* < 0.05.

**Table 4:** Engagement with mindfulness prospectively predicting mental health symptoms.

Next-Day Mental Health	*b* (SE)	*p*	95% CI
(Intercept)	1.13 (0.47) *	0.017	[0.21, 2.06]
M_Engagement	0.02 (0.05)	0.730	[−0.08, 0.11]
DEV_Engagement	0.06 (0.02) **	0.003	[0.21, 0.10]
Same-Day Mental Health	0.28 (0.03) ***	<0.001	[0.22, 0.35]
Next-Day Engagement	−0.07 (0.02) ***	<0.001	[−0.11, −0.03]
Age	−0.01 (0.02)	0.353	[−0.04, 0.02]
Baseline Mindfulness	−1.51 (0.58) *	0.010	[−2.64, −0.37]

M_Engagement represents the decomposed stable average of daily engagement with mindfulness, and DEV_Engagement represents intra-individual variability in daily engagement with mindfulness. *** *p* < 0.001, ** *p* < 0.01, * *p* < 0.05.

**Table 5:** Location scale model of daily engagement.

Daily Engagement	Estimate (SE)	*p*	95% CI
Between-Subjects Variance (α)			
(Constant)	0.05 (0.10)	0.841	[−0.40, 0.49]
M_Mental Health	−0.81 (0.36) *	0.025	[−1.52, −0.10]
DEV_Mental Health	−0.35 (0.12) **	0.003	[−0.58, −0.12]
Age	−0.24 (0.08) **	0.003	[−0.40, −0.08]
Baseline Mindfulness	−7.33 (2.13) **	0.001	[−11.50, −3.15]
Within-Subjects Variance (τ)			
(Constant)	−0.17 (0.89)	0.845	[−1.92, 1.57]
M_Mental Health	0.36 (0.21) ^†^	0.088	[−0.05, 0.78]
DEV_Mental Health	0.06 (0.06)	0.366	[−0.07, 0.19]
Age	−0.04 (0.03)	0.126	[−0.09, 0.01]
Baseline Mindfulness	2.72 (1.26)	0.031	[0.25, 5.18]
Association			
Linear	0.91 (0.13) ***	<0.001	[0.66, 1.16]
Scale			
Sigma	0.41 (0.09) ***	<0.001	[0.23, 0.58]

M_Mental Health represents the decomposed stable average of daily mental health symptoms, and DEV_Mental Health represents intra-individual variability in daily mental health symptoms. *** *p* < 0.001, ** *p* < 0.01, * *p* < 0.05, ^†^ *p* < 0.10.

**Table 6:** Location scale model of mental health.

Daily Mental Health	Estimate (SE)	*p*	95% CI
Between-Subjects Variance (α)			
(Constant)	−0.31 (2.25)	0.890	[−4.72, 4.10]
M_Engagement	−0.00 (0.20)	0.999	[−0.39, 0.39]
DEV_Engagement	0.14 (0.05) **	0.006	[0.04, 0.24]
Age	−0.01 (0.08)	0.845	[−0.16, 0.13]
Baseline Mindfulness	−1.55 (2.23)	0.486	[−5.91, 2.81]
Within-Subjects Variance (τ)			
(Constant)	−1.45 (0.72) **	0.042	[−2.84, −0.05]
M_Engagement	0.10 (0.07)	0.146	[−0.03, 0.23]
DEV_Engagement	−0.02 (0.04)	0.524	[−0.09, 0.05]
Age	0.00 (0.02)	0.998	[−0.04, 0.04]
Baseline Mindfulness	0.87 (0.89)	0.326	[−0.87, 2.61]
Association			
Linear	0.09 (0.09)	0.305	[−0.09, 0.27]
Scale			
Sigma	0.54 (0.07) ***	<0.001	[0.40, 0.68]

M_Engagement represents the decomposed stable average of daily engagement with mindfulness, and DEV_Engagement represents intra-individual variability in daily engagement with mindfulness. *** *p* < 0.001, ** *p* < 0.001.

## Data Availability

The raw data supporting the conclusions of this article will be made available by the authors upon request.
